# Ultra-Thin Terahertz Deflection Device Based on Laser Direct Writing Graphene Oxide Paper

**DOI:** 10.3390/mi13050686

**Published:** 2022-04-28

**Authors:** Yixin Suo, Luming Zhang, Yihang Li, Yu Wu, Jian Zhang, Qiye Wen

**Affiliations:** 1 Department of Electronic Science and Engineering, University Electronic Science and Technology of China, Chengdu 610054, China; 201922021714@std.uestc.edu.cn (Y.S.); lumingz@std.uestc.edu.cn (L.Z.); 201921020716@std.uestc.edu.cn (Y.L.); 202021020109@std.uestc.edu.cn (Y.W.); jianzhang@uestc.edu.cn (J.Z.); 2 Institute of Advanced Millimeter-Wave Technology, University Electronic Science and Technology of China, Chengdu 610054, China

**Keywords:** terahertz beam deflection, laser direct writing, graphene oxide, low reflection

## Abstract

In the world of terahertz bands, terahertz beam deflection has gradually attracted substantial attention, due to its great significance in wireless communications, high-resolution imaging and radar applications. In this paper, a low-reflection and fast-fabricated terahertz beam deflection device has been realized by utilizing graphene oxide paper. Using laser direct writing technology, graphene oxide has been patterned as a specific sample. The thickness of the graphene oxide-based terahertz devices is around 15–20 μm, and the processing takes only a few seconds. The experimental results show that the beam from this device can achieve 5.7° and 10.2° deflection at 340 GHz, while the reflection is 10%, which is only 1/5 of that of existing conventional devices. The proposed device with excellent performance can be quickly manufactured and applied in the fields of terahertz imaging, communication, and perception, enabling the application of terahertz technology.

## 1. Introduction

The terahertz band (0.1~10 THz) has already developed rapidly in the last two decades [1] and has been used in security imaging [2], biological detection [3], 6G communication [4] and other fields. In addition to a large number of terahertz functional devices, such as terahertz modulators [5,6] and terahertz absorbers [7,8,9], there is an increasing demand for THz beam control devices with beam steering and forming capabilities, especially terahertz wavefront modulation devices [10,11]. The phased array antennas were often used to achieve beam steering and forming. Due to the research barriers of the terahertz T/R module and the processing cost, it is difficult to manufacture a completed phased array that can be applied in the terahertz band. Although in the low terahertz frequency range (for example, 280 GHz [12]), CMOS-based phased arrays are manufactured using a complex fabrication process, and their performance is limited to a certain extent. By contrast, the binary Fresnel zone plate (FZP) has the characteristics of simple structure, easy manufacturing, and lower costs and assembly complexity, and this is why it has become an indispensable beam steering and imaging device in terahertz electronic systems [13,14,15,16]. In previous studies, antennas are based on silicon substrates [17], PCB-FZPs [18], LIG-FZPs [19] and other beam deflection devices, which are made of special materials such as liquid crystals [20,21,22]. These devices have the disadvantages of high reflection (FZPs’ ring belt materials, such as LIG and metal, and the reflection of a terahertz wave is greater than 85%), complex fabrication, and lack of flexibility. High reflection will interfere with the signal transmission, which leads to the decline of the instability. Moreover, the excessive reflection will affect the operation reliability and working performance of the excitation source. Many functional devices for terahertz band require photolithography to manufacture [12,18], which extends the period of fabricated production. Commonly used substrates cannot be bent enough and used in some terahertz integration systems with extreme demands, while flexible substrates can be adapted to these special circumstances. Following the above investigations, the low-reflection, easy-to-manufacture flexible terahertz beam control devices are promising for more frontier terahertz applications in terahertz wireless communication, imaging, and sensing.

Graphene oxide has an excellent transmission performance in terahertz band, and the transmission performance of graphene oxide decreases significantly after reduction treatment. Common reduction methods for graphene oxide include chemical reduction, thermal reduction, and laser reduction [23]. The laser direct writing equipment can reduce the specific shape of the laser-reduced graphene oxide on the surface of the graphene oxide. The whole process is simple and rapid, which is suitable for mass production. The method has been used in the preparation of flexible electrodes and supercapacitors [23,24,25,26]. At present, many graphene-based terahertz devices have been substantially developed [27,28]. The laser direct writing method based on graphene oxide can also be used to manufacture flexible terahertz beam deflection devices. The manufactured devices have low reflection, good flexibility, and high precision, which is convenient for the integration and assembly of the advanced terahertz system.

In this article, a low-reflection, ultra-thin flexible terahertz device, which is based on laser direct writing technology and graphene oxide paper, is proposed. The procedure for fabrication and the diffracting performance of the terahertz device are discussed in detail as follows.

## 2. Comparison between GO and LRGO

**Figure 1:**
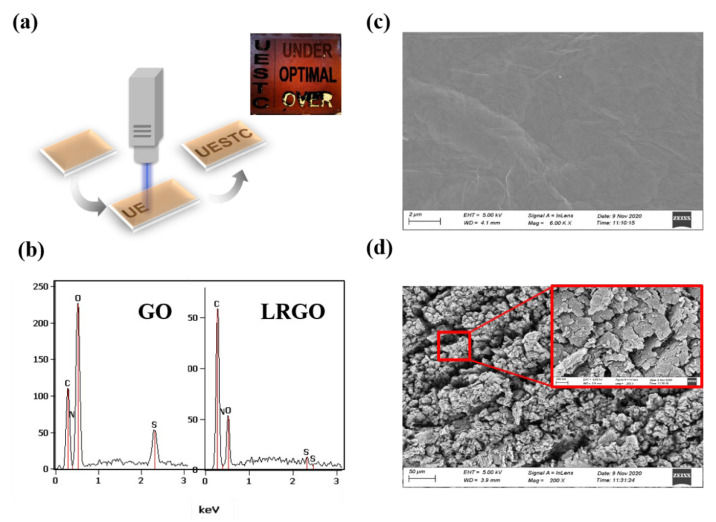
(**a**) Illustration of LRGO’s fabrication, inside of (**a**) is the photo of GO; over treatment−LRGO (20 times fabrication LRGO with scanning speed is 750 mm/s); optimal treatment−LRGO (15 times fabrication LRGO with scanning speed is 750 mm/s); under treatment−LRGO (5 times fabrication LRGO with scanning speed is 750 mm/s) and UESTC letter pattern. (**b**) EDS image of GO and LRGO. (**c**) SEM image of GO. (**d**) SEM image of 20 times fabrication LRGO.

Laser processing technology is a common method for manufacturing graphene-based electrodes; it can rebuild the surface structure of the sample. The thickness of GO paper is about 14–16 μm. Laser direct writing (LDW) equipment is used to reduce the GO paper, as presented in Figure 1a. The processed GO paper, so-called laser reduced graphene oxide (LRGO), is black with a rough surface, as shown in Figure 1b. It can be found that lower power is required to achieve reduction under N_2_ atmospheric conditions [24] and that GO paper can be hard to burn up without oxygen. A laser with a wavelength of 350 nm is selected in this work. The laser’s scanning speed is 750 mm/s, laser power has a specific value and the diameter of laser spot is around 14 μm, which provides the possibility of fabricating fine patterns with high precision. When processing GO paper with different powers, the obtained LRGO will also have different characteristics. Owing to the insufficient power of the laser, it will lead to an incomplete reduction of the GO paper. Compared with the color of GO, the color of the treated film surface is light black. One side of the treated film surface is rough, and the properties of the untreated film on the other side have not changed. This incompletely reduced graphene oxide paper is called under treatment-LRGO (UT-LRGO). The surface of the fully reduced LRGO is rough and the color is darker than UT-LRGO. It is more obvious that the reverse side of the completely reduced LRGO has obvious processed patterns. The complete reduction takes place in an instant, and the color deepens darker, accompanied by a popping sound. (Attach file Videos S1 and S2). This completely reduced graphene paper is called optimal treatment-LRGO (OT-LRGO). OT-LRGO is the best choice when making the proposed graphene-based devices. If OT-LRGO continues to be laser processed, then OT-LRGO will become over treatment-LRGO. The over treatment-LRGO was broken and fragile [29]. The laser energy is determined by multiple parameters such as scanning speed, scanning frequency, and processing times. Compared with processing times, other parameters are not easy to quantify. Therefore, we prefer different processing times to express LRGO with different levels of reduction.

The mechanism of laser reduction is as follows: GO contains a great quantity of oxygen-containing functional groups (OCGs) and carbon–oxygen bonds. With the treatment of the laser, the C-O bonds are broken. Meanwhile, the hydrogen and the oxygen elements in OCGs of GO escape in the form of a carbon and oxygen ratio (C/O) are measured by using EDS as shown in Figure 1b. 

With the laser treatment, the C/O of the samples is greatly reduced, which leads to a rise in conductivity. Four−point probe technique measurement shows that the conductivity of GO and LRGO are 1.4 × 10^−1^ S/m and 2000 S/m, respectively. Here, a method with low power and multiple printing times is applied to treat the GO paper, which ensures a high-level reduction and minor damage in GO paper. The oxygen and hydrogen elements in GO paper escape in the form of gas, resulting in many micro holes in GO paper multilayers, which leads to the looser samples. Despite all of these, the coral-like structure of OT-LRGO has been attributed to absorb the terahertz wave, as shown in Figure 1d. With the increase of laser energy (by changing the scanning rate or frequency of the LDW, etc.), the coral-like structure of the OT-LRGO collapses, and the film will become fragile. Therefore, to obtain a stronger LRGO paper that absorbs terahertz waves, it is important to adopt an appropriate parameter.

**Figure 2:**
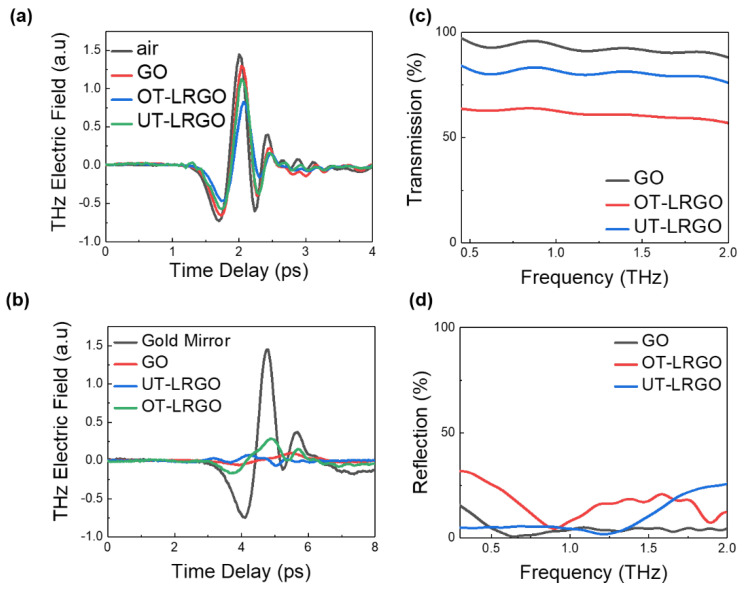
(**a**) Transmitted THz signals in the time domain of the air, GO, OT-LRGO, and UT-LRGO. (**b**) Reflected THz signals in the time domain of the Au, GO, and OT-LRGO (optimal treatment LRGO:15 times fabrication LRGO with scanning speed is 750 mm/s); UT-LRGO (5 times fabrication LRGO with scanning speed is 750 mm/s). (**c**) Transmission spectra of the GO, UT-LRGO, and OT-LRGO. (**d**) Reflection spectra of the GO, UT-LRGO, and OT-LRGO.

High conductivity and the coral-like structure reduce the overall transmission performance of the sample. The transmission of the sample was measured by the terahertz time-domain spectroscopy (THz-TDS) system, as shown in Figure 2a. Whether GO is treated with laser or not, the transmission response changes significantly. The transmission change of the sample, as well as computer-controlled laser writing, allows the film to be rapidly fabricated into terahertz steering devices. Taking air as a reference, the transmission of GO paper to terahertz wave reached 89.5%, while the transmission of LRGO was only 57.2%. Almost half of the transmission difference between LRGO and GO is the main factor that makes LRGO-based THz devices have lower insertion loss. Then, taking gold mirror as a reference, THz-TDS was used to measure the reflection of GO and LRGO. The results are shown in Figure 2d. After processing by LDW equipment, the reflection of GO remains at a low level and the transmission of GO decreases to some extent. 

In this measurement, the samples with different reduction of degrees were characterized by terahertz wave spectra. In Figure 2a,d, the GO films were processed 10 times (UT-LRGO) and 15 times (OT-LRGO) at a scanning speed of 720 mm/s, respectively.

The transmission and reflection of OT-LRGO are lower than that of UT-LRGO, because OT-LRGO has higher reduction, higher conductivity, and stronger ability to attenuate terahertz wave. The coral-like structure of OT-LRGO is mainly related to the decrease in the transmission of terahertz wave. LRGO has lower conductivity than metal, so LRGO has lower reflection than metal. Combining with our previous work [30], the main reason for the decrease in transmission is the ohmic loss of electromagnetic wave in the coral-like structure. Meanwhile, the transmission is more closely related to the degree of reduction.

## 3. LRGO-Terahertz-Device Design

**Figure 3:**
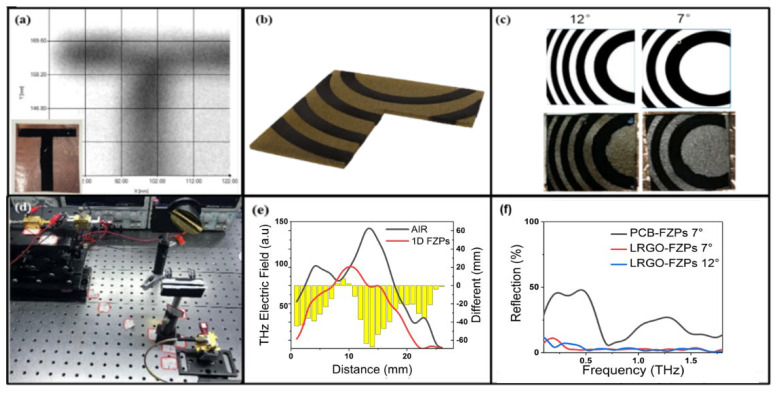
(**a**) LRGO-mask imaging results in terahertz band and the inset shows the image of the LRGO-mask. (**b**) Schematic of the LRGO-FZPs. (**c**) Calculated shape of FZPs and the photo of LRGO-FZPs processed by LDW. (**d**) THz communication verification system based on 340 GHz THz source. (**e**) Measured field-intensity distribution of 1D-FZPs. (**f**) Reflection spectra of the PCB-FZPs, LRGO-FZPs 7°, and LRGO-FZPs 12°.

Taking advantage of the different transmissions between the GO paper and the LRGO paper, as well as the low reflection characteristics of them, many terahertz band devices can be designed. Firstly, LRGO and GO have different terahertz transmissions and can be used to make flexible and low-reflection masks. Using LDW to process the GO paper into a T-shape pattern, a terahertz image can be obtained with the THz-TDS system. The resulting image contains a clear T-shape, as shown in Figure 3a.

Previous studies in beam steering devices based on FZPs can also be designed using GO/LRGO papers. In the proposed device, Fresnel Zone Plates (FZPs) have been designed for 340 GHz, with a focal length of 50 mm. According to WRC-19 [31], the working frequency of the device we proposed is a common frequency for communications. At 50 mm from the emitter, the Gaussian beam radius is approximately 7 mm, and the size of the FZPs plane has been set to 20 mm × 20 mm. Taking appropriate focal length (F) to aperture size (D) ratio (i.e., F/D ratio) and ratio α $(\alpha \;=\left(\mathrm{aperture}\;\mathrm{radius}/\mathrm{Gaussian}\;\mathrm{beam}\;\mathrm{radius}{)}^{2}\right)$ [11] can obtain a higher gain and efficiency of the proposed device. After determining the deflection angle and focal length, the center (C) and the radius (R) of the FZP patterns can be set up. Under the irradiation of a point light source, the phase distribution on the plane at a distance of focal length (50 mm) from the light source is given by $\varphi \left(r\right)=k\left(\sqrt{r^{2}+F^{2}}-F\right)$, where $k=\frac{2\pi }{\lambda }$, and λ is wavelength. When the beam is at the angle θ of the plane normal, the phase should be ${\;\varphi }_{d}\left(r\right)=\mathrm{krsin}\theta$. According to the phase difference between the φ(r) and $\varphi_{d}\left(r\right)$, we can calculate the center and the radius of the FZP patterns as follows:

(1) $$\;C_{n}=\frac{a_{n}sin\theta }{cos^{2}\theta }$$

(2) $$\;R_{n}=\frac{\sqrt{a_{n}^{2}-F^{2}+C_{n}^{2}cos^{2}\theta }}{cos\theta }$$

where $a_{n}=F-\frac{\left(\left(\varphi_{d}-\varphi \right)-n\pi \right)}{k}$. and the center and the radius of the FZP patterns are given in Table 1 at 340 GHz. 

In order to verify the validity of the center and radius of the calculated FZPs, a one-dimensional FZPs (1D-FZPs) with a 12-degree deflection has been designed [32]. The shape is similar to the grating structure. The parallel terahertz beam radiates on 1D-FZPs and scans the horizontal line of the beam center at a distance of 50 mm from FZPs. The results are shown in the Figure 3d. It can be seen that FZPs cause the terahertz wave to diffract and deflect significantly.

According to the formula and key parameters above, two-dimensional FZPs are processed by the laser direct writing (LDW) equipment (Attach file Video S3). When FZPs are processed, nitrogen is quantified to reduce the oxygen effect and the energy required for reduction. As a higher energy laser will quickly break through the GO paper, the fabrication uses smaller energy processing several times. The processed FZPs are shown in Figure 3c. These patterns are made by adopting a single-sided process, so it can be guaranteed that the pattern will not be deformed. The accuracy and completeness of the processed devices mainly depend on the selection of processing power and times. In addition to the broken devices mentioned above, excessive processing times will also cause the spread of the reductive pattern.

When imaging the device using terahertz wave, we can see that the LRGO patterns will reduce the transmission of the terahertz wave. From the previous demonstration, the reflection of both LRGO and GO are at a low level, so compared to the FZPs of PCB, LRGO-FZPs theoretically have a lower reflection. We used THz-TDS to test the PCB-FZPs and LRGO-FZPs, respectively, and the results are shown in Figure 3e. The PCB-FZPs reflection is greatly reduced compared with the metal plate, but it still maintains a high level. The coverage ratio of copper on the surface of PCB-FZPs is similar to the ratio of reflection intensity between PCB-FZPs and metal plates (45%). By contrast, the reflection of LRGO-FZPs is greatly reduced. LRGO-FZPs have the same pattern as PCB-FZPs. The reflection of LRGO-FZPs is only 1/5 of that of PCB-FZPs. This device has lower reflection and is more suitable for using in terahertz-integrated systems.

**Figure 4:**
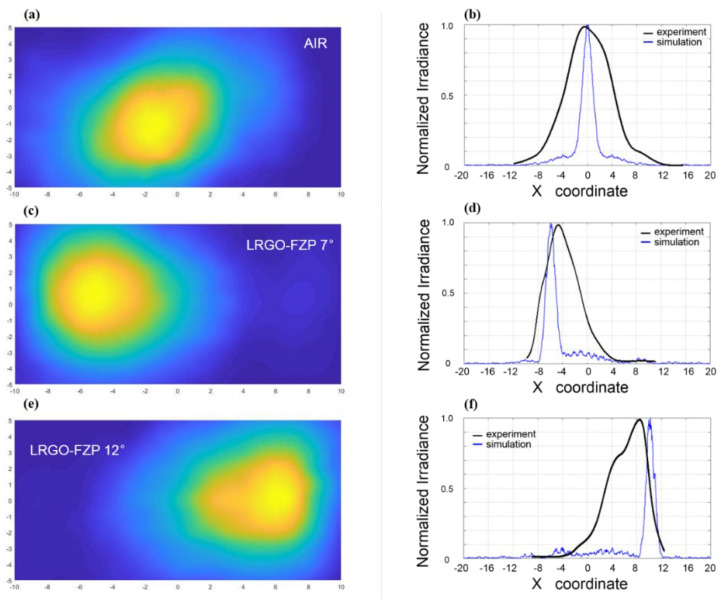
Measured THz field distributions on the focal plane, corresponding to the FZPs with air and different deflections of (**a**) air (**c**) 7°, and (**e**) 12°. Measured field-intensity distribution of the X-axis on the focal plane, corresponding to the FZPs with air and different deflections of (**b**) air, (**d**) 7°, (**f**) 12°.

The measured system, what we used, to verify the performance of FZPs is shown in Figure 4a. The terahertz beam with a central frequency of 340 GHz was originally generated by a Schottky diode multiplier (Virginia Diodes Inc. Charlottesville, VA) and coupled to free space using a pyramidal horn antenna. A parallel beam was created by placing the phase center of the pyramidal horn antenna at the focal point of the off-axis parabolic mirror. The detector was installed on a two-dimensional translation platform, which can be moved along the X-axis and Y-axis. By moving along the two mutually orthogonal axes, we can scan the image of the plane at the distance of focal length from the FZPs plane. The zone plate was placed at the distance of 100 mm behind the off-axis parabolic mirror. The terahertz wave with a center frequency of 340 GHz was radiated from the antenna horn and was shaped by the off-axis parabolic mirror to become a parallel beam that radiated to the surface of the zone plate. The detector was 50 mm behind the zone plate. According to the Cartesian coordinate system, the beam was incident on the negative semi-axis of the X-axis, and the moving platform can move on the YOZ plane.

The image was scanned point by point with a step of 1 mm, and then the measured result was obtained, as shown in Figure 4a–f. To facilitate scanning without changing the scanning range, LRGO-FZPs 7° and LRGO-FZPs 12° were placed in opposite directions; that is, the beam was deflected to the left after LRGO-FZPs 7°, and the beam was deflected to the right after LRGO-FZPs 12°. According to the obtained image, the beam has been deflected obviously, and the degree of deflection of LRGO-FZPs 12° to the right was greater than that of LRGO-FZPs 7°. Comparisons of the experimental data with simulation data from Zemax OpticStudio were shown in Figure 4b,d,f. As the distance from the beam center of the free space, the beam center of the LRGO-FZPs 7° scanning image was offset 5 mm to the left, and the deflection angle was 5.7° (θ = arctan (offset/focal length) = 5.7°), The beam center of the LRGO-FZPs 12° scanning image was shifted by 9 mm to the left, and the deflection angle was 10.2°. There is a fixed gap between the experimental data and the simulated data, which was caused by the inability of LRGO to completely shield the terahertz wave. The weak shielding ability also led to the result that LRGO-FZPs did not achieve focusing ability in the simulation.

## 4. Conclusions

In conclusion, the experiment measured the transmission and reflection of GO and LRGO. It is found that both of them can maintain low reflection, while the transmissions between them have great distinction. Subsequently, the method of fast processing GO/LRGO film can quickly prepare terahertz functional devices and perform terahertz imaging on the masks and FZPs manufactured by this method, and it is verified that the use of GO and LRGO can produce low-reflection terahertz devices. This kind of GO/LRGO terahertz device made with LDW equipment not only has the characteristics of being ultra-thin, flexible, and low reflection, but also can be processed quickly, which is conducive to mass manufacturing. They can be widely used in terahertz systems.

## Figures and Tables

**Table 1:** Geometric parameters of the calculated FZPs in different deflection.

**θ**	0°		7°		12°	
n	$C_{n}$	$R_{n}$	$C_{n}$	$R_{n}$	$C_{n}$	$R_{n}$
1	0	6.66	6.19	6.73	10.72	6.88
2	0	9.43	6.25	9.54	10.82	9.75
3	0	11.58	6.3	11.71	10.92	11.97
4	0	13.40	6.36	13.55	11.01	13.86
5	0	15.02	6.41	15.19	11.11	15.52
6	0	16.48	6.47	16.67	11.20	17.04
7	0	17.84	6.52	18.05	11.30	18.45

## Data Availability

Not Applicable.

## Supplementary Materials

The following are available online at https://www.mdpi.com/article/10.3390/mi13050686/s1, Video S1: Fixed GO paper for multiple laser direct writing processing, Video S2: Without fixed GO paper for multiple laser direct writing processing, Video S3: FZP preparation on GO paper by laser direct writing technology.

## References

[1] Zhang X.C., Shkurinov A., Zhang Y. (2017). Extreme terahertz science. Nat. Photonics.

[2] Federici J., Schulkin B., Huang F., Gary D., Barat R., Oliveira F., Zimdars D. (2005). THz imaging and sensing for security applications—explosives, weapons and drugs. Semicond. Sci. Technol..

[3] Yan X., Yang M., Zhang Z., Liang L., Wei D., Wang M., Zhang M., Wang T., Liu L., Xie J. (2019). The terahertz electromagnetically induced transparency—like metamaterials for sensitive biosensors in the detection of cancer cells. Biosens. Bioelectron..

[4] Rappaport T.S., Xing Y., Kanhere O., Ju S., Madanayake A., Mandal S., Alkhateeb A., Trichopoulos G.C. (2019). Wireless Communications and Applications Above 100 GHz: Opportunities and Challenges for 6G and Beyond. IEEE Access.

[5] Wen Q.Y., Tian W., Mao Q., Chen Z., Liu W.W., Yang Q.H., Sanderson M., Zhang H.W. (2014). Graphene based All−Optical Spatial Terahertz Modulator. Sci. Rep..

[6] Wen Q., He Y., Yang Q., Yu P., Feng Z., Tan W., Wen T., Zhang Y., Chen Z., Zhang H. (2020). High−Performance Photo−Induced Spatial Terahertz Modulator Based on Micropyramid Silicon Array. Adv. Mater. Technol..

[7] Zheng Z., Luo Y., Yang H., Yi Z., Zhang J., Song Q., Yang W., Liu C., Wu P. (2022). Thermal tuning of terahertz metamaterial absorber properties based on VO_2_. Phys. Chem. Chem. Phys..

[8] Zheng Z., Zheng Y., Luo Y., Yi Z., Zhang J., Liu Z., Yang W., Yu Y., Wu X., Wu P. (2022). A switchable terahertz device combining ultra—wideband absorption and ultra—wideband complete reflection. Phys. Chem. Chem. Phys..

[9] Wu X., Zheng Y., Luo Y., Zhang J., Yi Z., Wu X., Cheng S., Yang W., Yu Y., Wu P. (2021). A four−band and polarization−independent BDS—based tunable absorber with high refractive index sensitivity. Phys. Chem. Chem. Phys..

[10] Dorfmüller J., Vogelgesang R., Khunsin W., Rockstuhl C., Etrich C., Kern K. (2010). Plasmonic Nanowire Antennas: Experiment, Simulation, and Theory. Nano Lett..

[11] Hu D., Wang X., Feng S., Ye J., Sun W., Kan Q., Klar P.J., Zhang Y. (2013). Ultrathin Terahertz Planar Elements. Adv. Opt. Mater..

[12] Sengupta K., Hajimiri A. (2012). A 0.28 THz power-generation and beamsteering array in CMOS based on distributed active radiators. IEEE J. Solid-State Circuits.

[13] Zhao L., Duan W.H., Yelin S.F. (2011). All—optical Fresnel lens in coherent media: Controlling image with image. Opt. Express.

[14] Wang X., Xie Z., Sun W., Feng S., Cui Y., Ye J., Zhang Y. (2013). Focusing and imaging of a virtual all-optical tunable terahertz Fresnel zone plate. Opt. Lett..

[15] Avayu O., Eisenbach O., Ditcovski R., Ellenbogen T. (2014). Optical metasurfaces for polarization-controlled beam shaping. Opt. Lett..

[16] Huang K., Qin F., Liu H., Ye H., Qiu C.-W., Hong M., Luk’Yanchuk B., Teng J. (2018). Planar Diffractive Lenses: Fundamentals, Functionalities, and Applications. Adv. Mater..

[17] Shams I.B., Jiang Z., Rahman S.M., Cheng L.-J., Hesler J.L., Fay P., Liu L. (2017). A 740-GHz Dynamic Two-Dimensional Beam-Steering and Forming Antenna Based on Photo—Induced Reconfigurable Fresnel Zone Plates. IEEE Trans. Terahertz Sci. Technol..

[18] Hristov H.D. (2011). Terahertz Harmonic Operation of Microwave Fresnel Zone Plate Lens and Antenna: Frequency Filtering and Space Resolution Properties. Int. J. Antennas Propag..

[19] Wang Z., Wang G., Liu W., Hu B., Liu J., Zhang Y., Guang L. (2020). Patterned laser−induced graphene for terahertz wave modulation. J. Opt. Soc. Am. B.

[20] Solyankin P.M., Esaulkov M.N., Chernykh I.A., Kulikov I.V., Zanaveskin M.L., Kaul A.R., Makarevich A.M., Sharovarov D.I., Kameshkov O.E., Knyazev B.A. (2018). Terahertz Switching Focuser Based on Thin Film Vanadium Dioxide Zone Plate. J. Infrared Millim. Terahertz Waves.

[21] Hristov H.D., Rodriguez J.M., Grote W. (2012). The grooved-dielectric Fresnel zone plate: An effective terahertz lens and antenna. Microw. Opt. Technol. Lett..

[22] Scherger B., Reuter M., Scheller M., Altmann K., Vieweg N., Dabrowski R., Deibel J.A., Koch M. (2012). Discrete Terahertz Beam Steering with an Electrically Controlled Liquid Crystal Device. J. Infrared Millim. Terahertz Waves.

[23] You R., Liu Y., Hao Y., Han D., Zhang Y., You Z. (2020). Laser Fabrication of Graphene−Based Flexible Electronics. Adv. Mater..

[24] Wen F., Hao C., Xiang J., Wang L., Hou H., Su Z., Hu W., Liu Z. (2014). Enhanced laser scribed flexible graphene−based micro−supercapacitor performance with reduction of carbon nanotubes diameter. Carbon.

[25] Yang D., Bock C. (2017). Laser reduced graphene for supercapacitor applications. J. Power Sources.

[26] dos Júnior A.G.A., de Cardoso G.P., Paterno L.G., Ceschin A.M. (2020). Laser Reduction of Graphene Oxide/Zinc Oxide Nanoparticle Nanocomposites as a One−Step Process for Supercapacitor Fabrication. Phys. Status Solidi A-Appl. Mater. Sci..

[27] Kong X.-T., Khan A., Kidambi P.R., Deng S., Yetisen A.K., Dlubak B., Hiralal P., Montelongo Y., Bowen J., Xavier S. (2015). Graphene−Based Ultrathin Flat Lenses. ACS Photonics.

[28] He Y.-L., Liu J.-B., Wen T.-L., Yang Q.-H., Feng Z., Tan W., Li X.-S., Wen Q.-Y., Zhang H.-W. (2018). Flexible terahertz modulators based on graphene FET with organic high−k dielectric layer. Mater. Res. Express.

[29] Currie M., Caldwell J.D., Bezares F.J., Robinson J., Anderson T., Chun H., Tadjer M. (2011). Quantifying pulsed laser induced damage to graphene. Appl. Phys. Lett..

[30] Shui W., Li J., Wang H., Xing Y., Li Y., Yang Q., Xiao X., Wen Q., Zhang H. (2020). Ti3C2Tx MXene Sponge Composite as Broadband Terahertz Absorber. Adv. Opt. Mater..

[31] Marcus M.J. (2019). ITU WRC-19 Spectrum Policy Results. IEEE Wirel. Commun..

[32] Eisenbach O., Avayu O., Ditcovski R., Ellenbogen T. (2015). Metasurfaces based dual wavelength diffractive lenses. Opt. Express.

